# A Hardware Accelerator for Real-Time Processing Platforms Used in Synthetic Aperture Radar Target Detection Tasks

**DOI:** 10.3390/mi16020193

**Published:** 2025-02-07

**Authors:** Yue Zhang, Yunshan Tang, Yue Cao, Zhongjun Yu

**Affiliations:** 1 Aerospace Information Research Institute, Chinese Academy of Sciences, Beijing 100094, China; zhangyue20@mails.ucas.ac.cn (Y.Z.);; 2 School of Electronic, Electrical and Communication Engineering, University of Chinese Academy of Sciences, Beijing 101408, China

**Keywords:** synthetic aperture radar (SAR) imagery, convolutional neural networks (CNNs), target detection tasks

## Abstract

The deep learning object detection algorithm has been widely applied in the field of synthetic aperture radar (SAR). By utilizing deep convolutional neural networks (CNNs) and other techniques, these algorithms can effectively identify and locate targets in SAR images, thereby improving the accuracy and efficiency of detection. In recent years, achieving real-time monitoring of regions has become a pressing need, leading to the direct completion of real-time SAR image target detection on airborne or satellite-borne real-time processing platforms. However, current GPU-based real-time processing platforms struggle to meet the power consumption requirements of airborne or satellite applications. To address this issue, a low-power, low-latency deep learning SAR object detection algorithm accelerator was designed in this study to enable real-time target detection on airborne and satellite SAR platforms. This accelerator proposes a Process Engine (PE) suitable for multidimensional convolution parallel computing, making full use of Field-Programmable Gate Array (FPGA) computing resources to reduce convolution computing time. Furthermore, a unique memory arrangement design based on this PE aims to enhance memory read/write efficiency while applying dataflow patterns suitable for FPGA computing to the accelerator to reduce computation latency. Our experimental results demonstrate that deploying the SAR object detection algorithm based on Yolov5s on this accelerator design, mounted on a Virtex 7 690t chip, consumes only 7 watts of dynamic power, achieving the capability to detect 52.19 512 × 512-sized SAR images per second.

## 1. Introduction

Synthetic aperture radar (SAR) has become an essential observation tool in the field of remote sensing due to its all-weather, all-day capability and high resolution. In recent years, with technological advancements, SAR has played a crucial role in areas such as ocean and land monitoring [1]. Rapidly detecting specific targets during surveillance, particularly efficiently screening target objects from a vast amount of SAR data, holds significant importance.

However, traditional SAR target detection algorithms [2,3,4,5,6,7,8], such as those based on Constant False Alarm Rate (CFAR) methods, often rely on pre-set parameters. This approach heavily depends on estimating clutter in the scene and tends to lack good generalization across different scenarios. Therefore, in recent years, researchers have started to introduce new methods (such as the You Only Look Once (YOLO) series in deep neural networks) for SAR target detection and have achieved promising results [9,10,11,12,13,14]. Yang et al. [9] introduced a coordinate attention module (CoAM) to mitigate the disturbance from complex backgrounds. An improved YOLO v5-based azimuth-sensitive object detection method is proposed for such objects in SAR images that are azimuth-sensitive and of different scales by Ge [10]. In order to reduce computational time with relatively competitive detection accuracy, Guo et al. [13] develop a new architecture with a smaller number of layers called YOLOv2-reduced. But, while SAR target detection algorithms based on YOLO have improved detection performance, they also come with a significant computational burden. The substantial computational workload often leads to slower detection speeds.

To be able to respond quickly to situations, achieving rapid detection while maintaining a certain level of accuracy has become a new requirement. Therefore, numerous studies [15,16,17,18,19] are now focused on reducing computational load in detection algorithms to improve detection speed. Zhou et al. [15] proposed a hybrid representation learning-enhanced SAR target detection algorithm based on the unique features of SAR images from a lightweight perspective called HRLE-SARDet. Jiang et al. [16] introduced YOLO-V4-light network to achieve rapid detection. Liu et al. [18] proposed a lightweight ship detection network based on the YOLOv4-LITE model. Yet, these studies typically validate their results using high-power GPUs (Graphics Processing Units) as the computational platform.

To further accelerate the speed of detection, conducting real-time imaging and detection directly on satellite-borne or airborne platforms has become one of the solutions for achieving rapid detection. Due to the higher requirements for power consumption and hardware radiation resistance in satellite-borne or airborne platforms, Field-Programmable Gate Array (FPGA) [20] is undoubtedly a better choice compared to GPUs for achieving real-time detection on these platforms. However, the development complexity of FPGA is higher than that of GPUs, typically requiring developers to have a thorough understanding of the algorithm’s operational steps and FPGA hardware resources. Therefore, developing an accelerator that fully utilizes the on-chip resources of FPGA to achieve rapid SAR target detection tasks remains a challenge.

In the field of optics, there have been many advancements in accelerating the YOLO algorithm using FPGA [21,22,23,24,25]. Liu et al. [21] proposed Tensor Train (TT) decomposition for compressing the YOLOv5 model. Montgomerie-Corcoran et al. [22] employed a streaming architecture design for our YOLO accelerators, implementing the complete model on-chip in a deeply pipelined fashion. Li et al. [23] proposed a novel 16-bit dynamic fixed-point number quantization method to map the object detection network YOLOv4-tiny into FPGA-based heterogeneous deep learning accelerators. In recent years, researchers have also introduced FPGA-based acceleration methods into the SAR field to accelerate target detection algorithms [26,27,28]. In the past, when accelerating convolution modules, accelerators usually adopted a parallel acceleration method in two dimensions: input channels and output channels. This method of dimension expansion is often inflexible, resulting in inefficient utilization of DSP resources in FPGAs during convolution operations.

In response to the above challenges, we designed an accelerator for SAR target detection tasks based on FPGA, suitable for real-time detection on airborne or satellite platforms. Our main contributions are as follows:

(1) A novel convolution calculation unroll pattern that expands convolution operations in three dimensions, fully leveraging computational resources and enhancing the accelerator’s processing capabilities.
(2) We propose a memory arrangement suitable for convolution operations, reducing the time consumed by memory reads during algorithm execution.
(3) A dataflow computing pattern suitable for FPGA, reducing the time intervals between each operation.

## 2. Structure

**Figure 1:**
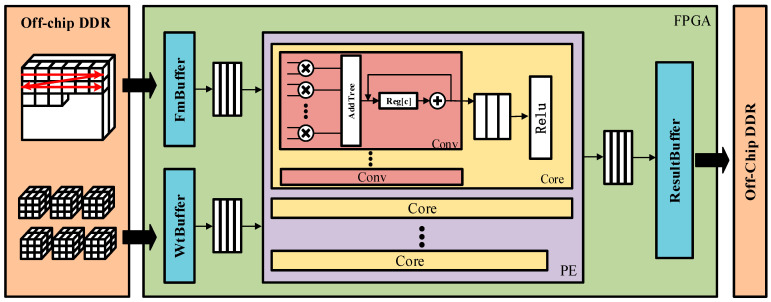
The structure of the entire accelerator.

This chapter introduces the structure of the entire accelerator shown in Figure 1. The platform is primarily divided into the off-chip Double Data Rate SDRAM (DDR) memory storage section and the on-chip FPGA computing section. For off-chip DDR storage, to enhance the platform’s bandwidth, we designed specialized memory arrangements for feature maps and weights to meet the requirements of continuous reading and writing. In the on-chip FPGA computing section, we implemented data buffers and a processing engine (PE). The buffers include FmBuffer, WtBuffer, and ResultBuffer. The functions of these buffers is to coordinate data transfer between DDR and PE to enhance performance. The processing engine is responsible for convolution calculations. We unroll the convolution operation from multiple dimensions and perform parallel computations to enhance the processing speed.

## 3. Methods

### 3.1. Multidimensional Parallel Computing Convolution Module

In SAR target detection algorithms based on deep learning, there is a significant amount of convolution computation involved. Convolution operations entail a large number of multiplication and addition calculations, which, when executed sequentially, can consume a considerable amount of time. To effectively harness the on-chip computing resources of FPGA and reduce the computational time of convolutions, researchers typically unfold or pipeline the nested multiplicative and additive operations in convolution across different dimensions. In this paper, we expand computations across three dimensions: the depth of input feature maps, the sliding window dimension of convolution operations, and the depth of output feature maps. These three dimensions are, respectively, represented as DimN, DimR, and DimM.

**Figure 2:**
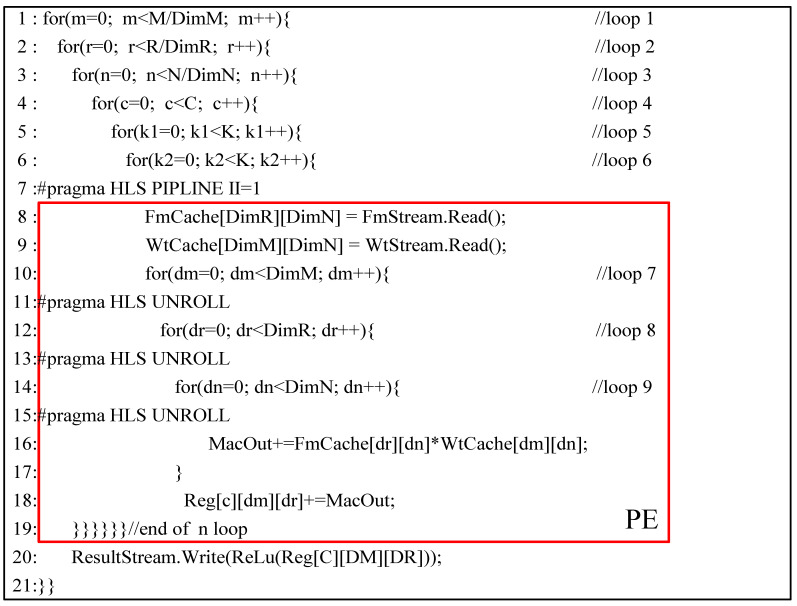
The specific breakdown of the convolution operation.

The specific breakdown of the convolution operation is shown in Figure 2. We divide the entire convolution computation into nine layers of loops. For the overall computation, accumulation operations need to be performed in loop3, loop5, loop6, and loop9, while the intermediate results calculated in loop4, loop7, and loop8 need to be stored in corresponding registers for later accumulation. Finally, the output is generated after the completion of loop3 calculations. Subsequently, computations proceed with loop1 and loop2 until the entire operation is finished.

**Figure 3:**
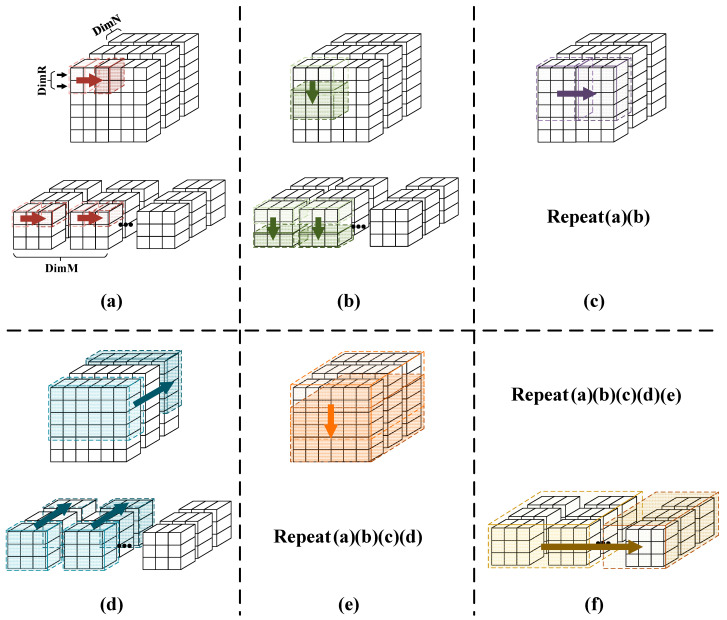
The data input order of the PE. (**a**) The data input corresponding to Loop6. (**b**) The data input corresponding to Loop5. (**c**) The data input corresponding to Loop4. (**d**) The data input corresponding to Loop3. (**e**) The data input corresponding to Loop2. (**f**) The data input corresponding to Loop1.

The PE module we designed completes the convolution operation between DimR×DimN feature maps and DimM×DimN weights in one clock cycle, corresponding to the loop7, loop8, and loop9 layers. Therefore, in each clock cycle, we only need to input DimR×DimN feature map data and DimM×DimN weight data to the PE. The specific data input to the PE are shown in Figure 3. We have segmented the input process into 6 stages, corresponding to the 6 layers of loops from loop1 to loop6 in Figure 2.

**Figure 4:**
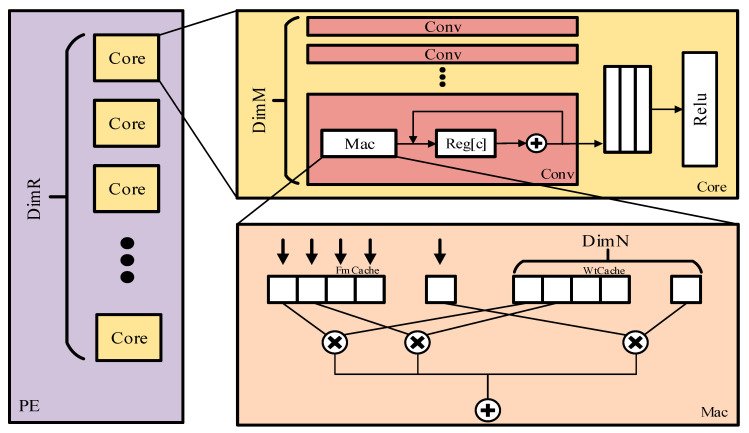
The hardware structure of the PE.

To implement the convolution computation shown in Figure 3, we designed the hardware structure of the PE as depicted in Figure 4. The PE is divided into three hierarchies based on the parallel dimension expansion method. These three levels are the PE unit, Core unit, and Mac unit. The fundamental computational unit is the Mac unit, which includes DimN FmCache units, WtCache units, DimN multipliers, and one adder. It is primarily responsible for computations in loop9. Above the Mac unit is the Core unit. The Core unit comprises DimM Conv units and one Relu unit. In each Conv unit, after the Mac unit completes a calculation, the intermediate result is stored in a register. During the execution of loops 3, 5, and 6, the adder in the Conv unit is used to accumulate the intermediate results until the final result is obtained. Then, once accumulation is complete, the final result generated by the Conv unit is output to a FIFO. Subsequently, the Relu unit reads data from the FIFO, performs activation operations, and generates the output. This process is repeated to complete operations for loop1 and loop2. The top layer is the PE unit. The PE consists of DimR Core units, allowing these units to execute the mentioned operations in parallel.

### 3.2. High Utilization Rate Memory Arrangement

**Figure 5:**
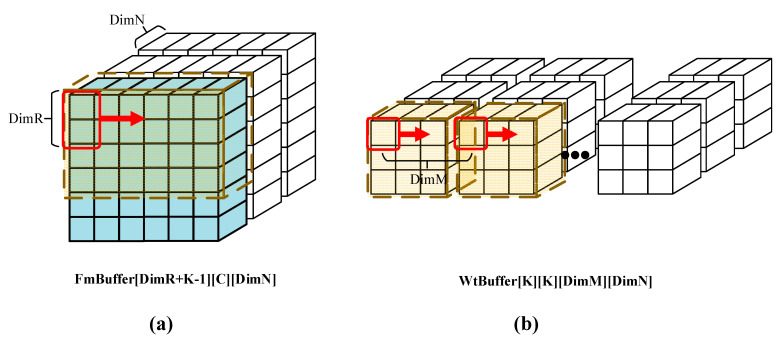
The struct of Buffer. (**a**) The struct of FmBuffer. (**b**) The struct of WtBuffer.

Due to limited on-chip storage resources, significant amounts of data are stored off-chip in DDR memory, necessitating staged loading into on-chip buffers. However, each time data are fetched from DDR to the buffer, it incurs a latency of several clock cycles to initiate the memory controller. Frequent small-scale data fetches can lead to inefficiencies in data retrieval. Therefore, to address this issue, this paper proposes a data arrangement pattern that facilitates high-contiguity reads. The sorting methods for feature map and weight data are illustrated in Figure 5.

**Figure 6:**
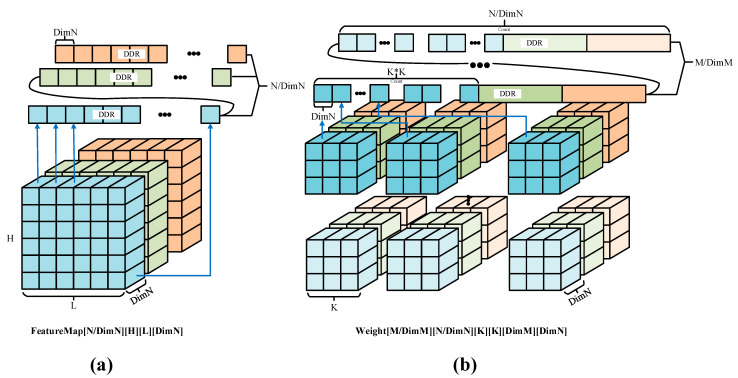
The memory arrangements. (**a**) The memory arrangement of FeatureMap. (**b**) The memory arrangement of Weight.

Figure 6 shows the arrangement of data in DDR. It is divided into two parts. Part (a) shows the arrangement of the Feature Map, and part (b) shows the arrangement of the Weight. For the feature map data, the original storage format was likely in three dimensions, as FeatureMap [H][L][N]. In this paper, the depth direction of FeatureMap is divided into segments based on DimN, resulting in a rearranged storage format of FeatureMap [N/DimN][H][L][DimN]. During storage, the process involves storing DimN data points first along the depth direction, then along the L direction storing H×DimN data points, followed by storing L×H×DimN data points along the H direction, and finally repeating these steps to store the rest of the divided data.

In the case of Weight data, the original storage format was Weight [M][K][K][N]. Building upon this format, the first dimension N and the fourth dimension M are partitioned. The final storage format becomes Weight [M/DimM][N/DimN][K][K][DimM] [DimN]. During the storage process, DimN data are initially stored along the depth direction, followed by storing DimM×DimN data points along the DimM direction, representing DimM different convolutional kernels’ DimN data points. Subsequently, storage occurs along the horizontal direction K1 and the vertical direction K2 of the convolutional kernel, followed by repeating these steps to store data in the divided depth direction for the DimM kernels. Finally, the process reiterates the initial steps to store the data for the remaining divided kernels.

### 3.3. The Streaming Computing Mode Suitable for Convolution

To perform a complete convolution calculation, a series of processes, including FmBufferIn(), FmBufferOut(), WtBufferIn(), WtBufferOut(), Process(), ResultIn(), and ResultOut(), are required. FmBufferIn() and WtBufferIn() involve transferring feature map data and weight data from off-chip DDR to on-chip buffers. FmBufferOut() and WtBufferOut() pertain to transferring feature map data and weight data from on-chip buffers to the PE calculation module. Process() denotes the calculation process within the PE. ResultIn() and ResultOut() represent data transfer from the PE to ResultBuffer and from ResultBuffer back to the off-chip DDR, respectively.

**Figure 7:**
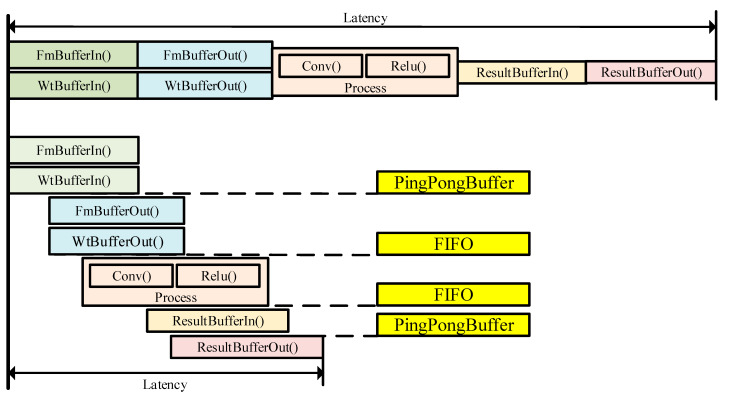
The streaming computing mode.

Due to data dependencies, these processes often need to be executed sequentially, leading to extended computation times. As shown in Figure 7, to abbreviate the overall computation duration, this paper employs PingPong buffers or FIFO buffers to interconnect between these processes. By integrating buffers, as soon as one process generates a portion of data, the following process can begin execution, without the necessity of waiting for the completion of data production in the previous process. This methodology effectively reduces latency.

## 4. Experimental Results

### 4.1. Platform

In this paper, we utilized two computing platforms. The first platform was NVIDIA’s 2080Ti, running on a Ubuntu 20.04 LTS operating system, equipped with computing tools such as Python 3.8, PyTorch 1.11.0, CUDA 11.3, and cuDNN 8.6.0. This platform serves a dual purpose: training SAR target detection networks and a comparison with the accelerator proposed in this paper. The other platform is an FPGA platform used to implement the accelerator described in this paper. We chose Xilinx’s Virtex-7 690t chip as the core processor and designed a dedicated processing board based on this chip, which includes the selected FPGA chip and an external 4GB DDR memory unit.

### 4.2. Dataset

This paper uses the widely adopted SSDD dataset, which has been embraced by numerous scholars since its release. The dataset is annotated in PASSCAL VOC format and divided into training, testing, and validation sets at a ratio of 7:2:1, thereby possessing the characteristics of a standard dataset. Moreover, from the perspective of SAR features, this dataset covers SAR images with various polarizations, simple backgrounds (open sea), and complex backgrounds (coastal areas), and includes SAR targets of different scales, enabling thorough validation of detector performance and detection platform capabilities.

### 4.3. Model

To verify the performance of the accelerator, this paper selects two parameters and relatively lightweight detection models. These two models are yolov5n and yolov5s. The parameter size and computational load of each detection network are shown in the Table 1 below.

### 4.4. Results

All of the experimental results are divided into two parts. In the first part, we demonstrate the detection accuracy and speed of the four models on the FPGA platform and compare them with the results obtained from training on the GPU platform, showcasing the performance of the accelerator. In the second part, we compare the detection accuracy, detection speed, and power consumption of our study’s model with those of other scholars, displaying the superiority of the proposed accelerator.

#### 4.4.1. Performance Results

In this study, the two lightweight detection networks mentioned above were deployed on the accelerator proposed in this paper, and the detection results (latency, accuracy, and dynamic power consumption) are shown in the Table 2. In the experiments, we tested using images of size 512 × 512.

**Figure 8:**
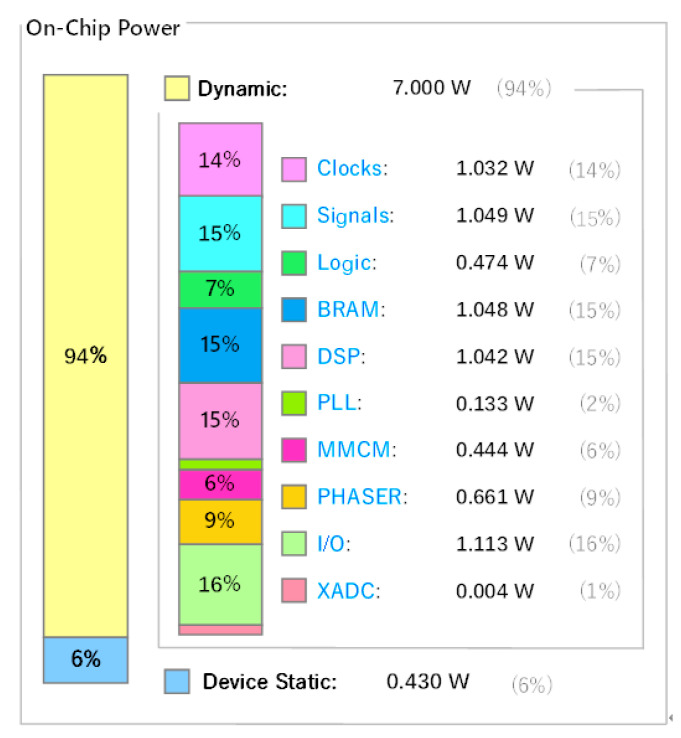
The figure of dynamic power.

In evaluating detection accuracy, prior to deploying the SAR object detection algorithm on the accelerator introduced in this paper, we initially quantized the network to 8 bits. This process resulted in a decrease in detection accuracy when running on the accelerator, as seen in the 5th column of the table. In addition, to validate the computational efficiency of the accelerator under effective power consumption, we introduced the latency-power product as a benchmark. For power consumption, we used the dynamic power consumption of the FPGA chip, and the specific values are shown in Figure 8. This metric represents the product of the computational latency required to complete the SAR object detection task and the corresponding dynamic power consumption (the power difference between running the SAR object detection algorithm and not running it). A lower latency–power product indicates that the system can accomplish tasks with decreased power consumption or complete them more rapidly within a given power budget. We conducted tests using the existing YOLOv5 architecture on an NVIDIA GeForce RTX 2080 Ti graphics card and compared the results with those presented in this paper. From the last column of the table, it is evident that the latency–power product of the proposed accelerator significantly surpasses that of the 2080Ti. When running the SAR object detection algorithm based on YOLOv5, achieving a detection accuracy of 93.11%, it simultaneously attains the capability to detect 52.19 SAR images of size 512 × 512 per second at a power consumption of 7.0 watts.

#### 4.4.2. Comparison Results

In this paper, the performance of the FPGA accelerator proposed here is compared with that of other FPGA accelerators applied to the YOLO algorithm. The comparison results are shown in the following table. Since the platforms, algorithms, and sizes used by the accelerators are different, we use GOPS/DSP as the final performance measurement standard. This indicator can objectively reflect the computing power of the processor. GOPS (Giga Operations Per Second) represents the throughput of the processor, and its calculation method is as follows:

(1) GOPS=GOP/Latency

Here, the abbreviation GOP (Giga Operation) refers to the computational complexity of the model for inferring a single image.

As can be seen from the data in the Table 3, compared with the works in References [21,22] that also use the YOLOv5s model, the throughput of the accelerator proposed in this paper are greatly improved, with increases of 92% and 27%, respectively. Moreover, even when different processing platforms are adopted, the GOPS/DSP indicators of the accelerator proposed in this paper are 0.27 and 0.08 higher than those in References [21,22], respectively. When comparing our results with the works in References [24,25] that use different models and platforms, we can see from the GOPS/DSP indicator alone that the DSP utilization efficiency of the accelerator proposed in this paper is 57%, 36%, and 328% higher than that of these three works, respectively. This proves the advancement of the work in this paper.

Table 4 presents a comparative analysis between the synthetic aperture radar (SAR) target detection algorithm accelerator proposed in this paper and those designed by previous researchers. It is clearly evident from the last column of the table that the accelerator proposed in this paper can perform 536.09 calculations per second, demonstrating superior computational performance compared to the accelerators proposed by previous researchers. When compared with the accelerators in References [26,27], the computational speed of the accelerator designed in this paper is 6.22 times and 7.85 times faster, respectively. Notably, the authors of Reference [28] optimized the detection model in YOLOv2, reducing the computational load of the model to 0.56 g and achieving an excellent result of up to 653 frames per second (FPS). However, the computational capacity of their accelerator is only 366 GOPS, which is lower than that of the accelerator designed in this study. In conclusion, the accelerator proposed in this paper has significant computational advantages.

## 5. Conclusions

This article presents a low-power, low-latency deep learning SAR target detection algorithm accelerator designed to achieve real-time target detection on airborne and satellite platforms. Initially, a processing engine suitable for multi-dimensional convolution parallel computing is proposed to fully utilize FPGA computation resources and reduce convolution computation time. Additionally, a unique memory arrangement is designed based on this processing engine to enhance memory read/write efficiency. Furthermore, a data flow pattern suitable for FPGA computation is implemented in the accelerator to reduce latency in each computation. In the end, we tested YOLOv5s on the accelerator and achieved a latency–power product as low as 134.12. This outcome indicates that when running the SAR target detection algorithm based on YOLOv5s on this accelerator, with a dynamic power of 7W, only 7.11 milliseconds are needed to detect a single 512 × 512 SAR image, meeting the low-power real-time processing requirements for airborne and satellite platforms.

## Figures and Tables

**Table 1:** The relevant information about the model.

Model	Input Size	Parameters	Flops
Yolov5n	640 × 640	1.9 M	4.5 g
Yolov5s	640 × 640	7.2 M	16.5 g

**Table 2:** Performance results (* = Multiplication sign).

Model	Platform	Input Size	Quantization	mAP(%)	Latency (ms)	FPS	Power	Latency * Power
Yolov5n	2080Ti	512 × 512	FP32	95.06	6.85	145.96	50.1	343.185
	Ours		A8W8	93.09	18.71	53.39	7.0	130.97
Yolov5s	2080Ti	512 × 512	FP32	95.62	7.11	140.65	50.4	358.34
	Ours		A8W8	93.11	19.16	52.19	7.0	134.12

**Table 3:** Comparison with other yolo accelerators.

Previous Work	Model	Platform	Input Size	Quantization	DSP	BRAM	LUT	GOPS	GOPS/DSP
[21]	Yolov5s	KCU116	640 × 640	--	1321	220	182k	42.6	0.03
[22]	Yolov5s	VCU110	640 × 640	A16W8	1794	1888	602k	392	0.22
[23]	Yolov4-Tiny	ZedBoard	416 × 416	A16W16	149	132	31K	0.4	0.003
[24]	Yolov3-Tiny	KU040	416 × 416	W16A16	1255	447	181K	233.6	0.19
	Yolov4-Tiny	KU040	416 × 416	W16A16	839	384	139K	181.4	0.22
[25]	Yolov3-Tiny	ZedBoard	416 × 416	W16A16	160	92.5	26K	10.5	0.07
Ours	Yolov5s	Virtex7 690T	512 × 512	A8W8	1786	1706	210K	536.09	0.30

**Table 4:** Comparison with YOLO accelerators in the SAR field.

Previous Work	Model	Dataset	Platform	Input Size	mAP(%)	FPS	GOP	GOPS
[26]	Efficient-YoloV3	Ship_Dataset	Nebula Accelerator X3	256 × 256	77.45	24.63	3.5	86.205
[27]	Yolov5s	Ship_Dataset	XCVU9P	256 × 256	78.74	25.86	2.64	68.27
[28]	MobileNet-Yolov2	SSDD	Virtex7 690T	416 × 416	93.3	653	0.56	366
Ours	Yolov5s	SSDD	Virtex7 690T	512 × 512	93.11	52.19	10.272	536.09

## Data Availability

Data are contained within the article.
